# Hypoxia Augments Cerebral Inflammation in a Dextran Sulfate Sodium-Induced Colitis Mouse Model

**DOI:** 10.3389/fncel.2020.611764

**Published:** 2020-12-09

**Authors:** Ying Han, Liping Ding, Xiang Cheng, Ming Zhao, Tong Zhao, Liang Guo, Xinyang Li, Yanan Geng, Ming Fan, Hong Liao, Lingling Zhu

**Affiliations:** ^1^Institute of Military Cognition and Brain Sciences, Academy of Military Medical Sciences, Beijing, China; ^2^Beijing Institute of Brain Disorders, Laboratory of Brain Disorders, Ministry of Science and Technology, Collaborative Innovation Center for Brain Disorders, Capital Medical University, Beijing, China; ^3^National Nanjing Center for Drug Screening, China Pharmaceutical University, Nanjing, China; ^4^Co-Innovation Center of Neuroregeneration, Nantong University, Nantong, China

**Keywords:** hypoxia, dextran sodium sulfate (DSS), colitis, neuroinflammation, microglia

## Abstract

The importance of hypoxia in the pathophysiology of inflammatory bowel disease (IBD) is increasingly being realized; also, hypoxia seems to be an important accelerator of brain inflammation, as has been reported by our group and others. IBD is a chronic intestinal disorder that leads to the development of inflammation, which is related to brain dysfunction. However, no studies have reported whether hypoxia is associated with IBD-induced neuroinflammation. Therefore, the objective of the present study was to determine whether hypoxia augments cerebral inflammation in a DSS-induced colitis mouse model. The mouse model was developed using 3% DSS for five days combined with exposure to hypoxic conditions (6,000 m) for two days. Mice were randomly divided into four groups: control group, DSS group, hypoxia group, and DSS plus hypoxia group. The results demonstrated that DSS combined with hypoxia resulted in up-regulation of colonic and plasmatic proinflammatory cytokines. Meanwhile, DSS plus hypoxia increased expression of Iba1, which is a marker of activated microglia, accompanied by increased expression of tumor necrosis factor-α (TNF-α), interleukin-1β (IL-1β), and interleukin-6 (IL-6) in the brain. Moreover, the expression of tight junction proteins, such as zonula occludens-1 (ZO-1), occludin, and claudin-5, was markedly downregulated. The current study provides new insight into how hypoxia exposure induces excessive inflammatory responses andpathophysiological consequences in the brain in a DSS-induced colitis model.

## Introduction

Inflammatory bowel disease (IBD), which is comprised of two main types, ulcerative colitis (UC) and Crohn’s disease (CD), is characterized by chronic or relapsing immune activation and inflammation within the gastrointestinal tract that markedly give rise to diarrhea, cramping, and pain, which are all standard symptoms of IBD (Wirtz et al., 2017). The pathogenesis of IBD is not fully understood, but hypoxia seems to be an important driver of inflammation (Vavricka et al., 2016). Gastrointestinal problems are a well-known complication during high-altitude activities. In a previous study, the authors investigated healthy volunteer mountaineers before and after an ascent to 4,500 m. On day 4, 61% of the volunteers showed endoscopic abnormalities (Vavricka et al., 2017). Studies have shown that high altitude exposure may lead to ulcer formation and gastroduodenal erosions, accompanied by subsequent gastrointestinal bleeding (Anand et al., 2006; Wu et al., 2007). These results imply that hypoxia exposure may have profound effects on the gastrointestinal tract. The role of hypoxia as an inducer of inflammation has also been studied. Several studies have shown that hypoxia exposure influences vessel permeability and inappropriate release of nitric oxide (Eckle et al., 2008; Wilson et al., 2009).

IBD leads to brain-mediated physiological and behavioral changes, and the underlying mechanisms may involve the immune response in the brain. 2,4,6-Trinitrobenzene sulfonic acid (TNBS)-treated adult male rats exhibited a marked inflammatory response in the brain that was characterized by microglial activation and TNF-α production, leading to increased susceptibility to seizures (Riazi et al., 2008). Furthermore, chronic intestinal inflammation alters hippocampal neurogenesis, which is also related to activated microglia and pro-inflammatory cytokines in the hippocampus of DSS-treated mice (Zonis et al., 2015). Our previous data also suggest that DSS-induced colitis exacerbates brain damage and augments microglial and inflammatory factor infiltration (Han et al., 2018).

A growing number of reports have also shown that hypoxia aggravates systemic inflammation-induced neuroinflammation and cerebral injury (Song et al., 2016). Hypoxia exposure (6,000 m, 6 h) augments lipopolysaccharide (LPS)-induced brain inflammation and induces the development of cerebral edema in mice at high altitudes (Zhou et al., 2017). Inflammatory reaction in the intestine also leads to systemic inflammation. When the gut is inflamed, there is a breakdown of the intestinal barrier function, and some theories posit that when the gut barrier is broken, circulating LPS levels and inflammatory factors are increased, inducing neuroinflammation (Kelly et al., 2015). A combination of risk factors for IBD seems to initiate alterations in epithelial barrier function, thereby allowing the translocation of LPS into the bowel wall (Neurath, 2014). Subsequently, aberrant and excessive cytokine responses cause brain inflammation.

Furthermore, some studies demonstrated that the increase in colonic inflammation is closely related to constitutive epithelial hypoxia-inducible factor (HIF) signaling (Tambuwala et al., 2010). Hypoxia and the upregulation of HIFs induced by hypoxia are detrimental to colon tissue and potentially exacerbate colitis. However, previous studies had shown HIF-1a as a critical regulator of barrier protection during DSS and TNBS-induced colitis in the early stage (Robinson et al., 2008; Shah et al., 2008). The latter study suggested that sustained colitis was mediated by the HIF-2a-induced activation of macrophage migration inhibitory factor (MIF; Shah et al., 2008). Growing evidence indicates that MIF functions as a proinflammatory cytokine and a hormone involved in inflammation-associated pathophysiology, which can increase microglia accumulation and activate inflammatory signaling in astrocytes (Su et al., 2017). Hypoxia can also amplify the NF-κB pathway by increasing the expression and signaling of Toll-like receptors (TLRs; Kuhlicke et al., 2007), which are extracellular receptors of bacterial LPS.

One way by which intestinal inflammation alters the function of the central nervous system (CNS) is through the disruption of the blood-brain barrier (BBB; Natah et al., 2005). The BBB is primarily comprised of endothelial cells that line the cerebral microvascular endothelial cells, which are surrounded by basement membranes, astrocytes, and pericytes (Takeshita and Ransohoff, 2012). At each site, the physical barrier is mainly regulated by tight junctions (TJs) that reduce the permeability of the intercellular adhesion areas (Engelhardt and Sorokin, 2009). The BBB isolates the brain parenchyma from circulating immune cells and antibodies. Some results suggested that hypoxia accelerates the LPS-induced disruption of BBB permeability in mice brain (Zhou et al., 2017). When BBB integrity is disrupted, a variety of inflammatory cytokines enter the brain and activate microglia.

The present study aimed to explore hypoxia-induced brain injuries in a colitis mouse model. We hypothesized that hypoxia aggravates the symptoms of colitis, triggering an inflammatory response in the brain and resulting in exacerbated brain damage. Therefore, we investigated the effects of hypoxia on the expression of proinflammatory cytokines in colonic tissues and serum. Furthermore, we explored the activation of microglia, secretion of inflammatory cytokines, and expression of tight junction proteins in the brain.

## Materials and Methods

### Mouse Models

Mice were purchased from the Laboratory Animal Center of Vital River Experimental Animal Company (Beijing, China). In all the experiments, the ethics guidelines for investigations involving conscious animals were followed, and the experiments were approved by the Animal Care and Use Committee of the Institute of Basic Medical Sciences (NO. IACUC-2017049). Seven- to eight-week-old male C57BL/6 mice weighing 18 ± 2 g were housed under a 12-h light/dark cycle with free access to standard rodent chow and water. The mice were maintained under specific-pathogen-free (SPF) conditions.

A well-characterized chemical model was used to initiate experimental IBD by giving the mice access to 3% DSS (MP Biomedicals, Santa Ana, CA, USA) in their drinking water for 5 days combined with exposure to hypoxia (6,000 m) for 2 days. The mice were divided into four groups (control, DSS, hypoxia, and DSS + hypoxia) and sacrificed on day 7 after the beginning of DSS administration in the experiments. Colonic tissue, brain tissue, and serum were collected for the experiments.

### Hematoxylin-Eosin (HE) Staining

The mice were anesthetized with 1% sodium pentobarbital by intraperitoneal injection and sacrificed. Then, the colons were isolated and sectioned. The colons were washed twice in pre-cooled PBS and soaked overnight in paraformaldehyde (4% in 0.1 mol/L phosphate buffer, pH 7.4). After immersion in absolute ethyl alcohol through a graded alcohol series, the tissues were embedded in paraffin, sectioned, and stained. Then, the colonic tissue sections were scanned by a Nanozoomer-XR Scanner C12000 (Hamamatsu Inc., Japan).

### Disease Activity Index (DAI)

To explore the effects of hypoxia on the development of DSS-induced colitis, the mice were individually evaluated daily to determine the body weight, stool consistency, and bleeding, as detailed in Table 1. Each parameter was assigned a score according to the previously proposed criteria and used to calculate the disease activity index (DAI) from day 0 to day 7 (da Silva et al., 2016; Bibi et al., 2017; Table 1).

**Table 1:** Disease activity index (DAI) score.

Score	Weight loss	Stool consistency	Bleeding
0	None	Normal	Normal
1	1–5%	–	–
2	5–10%	Loose stools	–
3	10–20%	–	–
4	>20%	Diarrhea	Gross bleeding

### Quantitative Real-Time PCR

Total RNA was isolated from mouse distal colon tissues using TRIzol reagent according to the manufacturer’s instructions (Invitrogen, Carlsbad, CA, USA). Reverse transcription was performed by a reverse transcription kit (Vazyme Biotech Company Limited, China). cDNA was amplified by real-time PCR using SYBR Green master mix (Genstar Biotech, China) as recommended by the manufacturer. Oligonucleotide primer sequences used for real-time PCR are in Table 2.

**Table 2:** Primer sequences used for real-time PCR.

Genes	Species	Sequences
TNF-α	Mouse	reverse:5′-TTGGTTAGCCACTCCTTC-3′
		forward:5′CCCTCACACTCAGATCATCTTCT-3′
IL-1β	Mouse	forward: 5′-TTCAGGCAGGCAGTATCACTC-3′
		reverse: 5′-GAAGGTCCACGGGAAAGACAC-3
IL-6	Mouse	forward: 5′-AGTCCTTCCTACCCCAATTTCC-3′
		reverse: 5′-GCTACGACGTGGGCTACAG-3′
β-actin	Mouse	forward: 5′-ACTGTCGAGTCGCGTCCA-3′
		reverse: 5′-GTCATCCATGGCGAACTGGT-3′

### Enzyme-Linked Immunosorbent Assay (ELISA)

Blood samples were clotted for 2 h at room temperature before centrifugation (4°C, 2,000 *g*, 30 min). TNF-α, IL-1β, and IL-6 in serum were measured using a mouse Quantikine ELISA kit (Neobioscience Technology Company Limited, Shenzhen, China) according to the manufacturer’s instructions.

### Immunohistochemical Staining

The mice were anesthetized with 1% sodium pentobarbital by intraperitoneal injection and perfused with prechilled saline (0.9%) to remove the circulating blood cells. The brains were washed twice with precooled PBS and soaked overnight in paraformaldehyde (4% in 0.1 mol/L phosphate buffer, pH 7.4) after each brain was dehydrated and frozen-sectioned at thicknesses of 20 μm. First, peroxidase was removed using 10% H_2_O_2_. Then, the sections were blocked with 5% BSA (in 1% PBST) for 30 min at 37°C and then incubated with a specific primary antibody (Iba-1,1:400, WAKO, Tokyo, Japan) overnight at 4°C before incubation with a secondary antibody (ZSGB-BIO, Beijing, China) for 90 min at 37°C. The color reaction was developed by incubation with a 3,3′-diaminobenzidine (DAB) solution for approximately 3 min. The sections were imaged under a microscope (Olympus, Tokyo, Japan).

### Western Blotting

The protein expression levels of ZO-1, occludin, and claudin-5 in the cortex tissues were determined. The samples were homogenized using RIPA lysis buffer on ice. Each sample was separated by SDS–PAGE. Then, the proteins were transferred to polyvinylidene difluoride membranes. After blocking, specific primary antibodies (ZO-1, 1:1,000, Invitrogen, CA, USA; occludin, 1:1,000, Invitrogen; claudin-5, 1:1,000, Thermo Fisher Scientific, Waltham, MA, USA; and β-actin, 1:10,000, Sigma) were incubated overnight at 4°C. After washing, the membranes were incubated with HRP-conjugated secondary antibodies (1:5,000, Bio-Rad, Hercules, CA, USA) for 1 h at room temperature. The specific bands were visualized using an ECL detection kit (Bio-Rad, Hercules, CA, USA). Quantification of the band intensities was performed by normalization to β-actin.

### Statistical Analysis

The data were analyzed using GraphPad Prism 7.0. The data are presented as the arithmetic mean ± SEM. Statistically significant differences between groups were determined using one-way ANOVA with Dunnett’s test. Two-way ANOVA followed by Bonferroni’s *post hoc* test was used for evaluating multiple comparisons. For all analyses, *p < 0.05, **p < 0.01 and ***p < 0.001 were considered statistically significant.

## Results

### Hypoxia Aggravated Colonic Inflammation Induced by DSS

**Figure 1:**
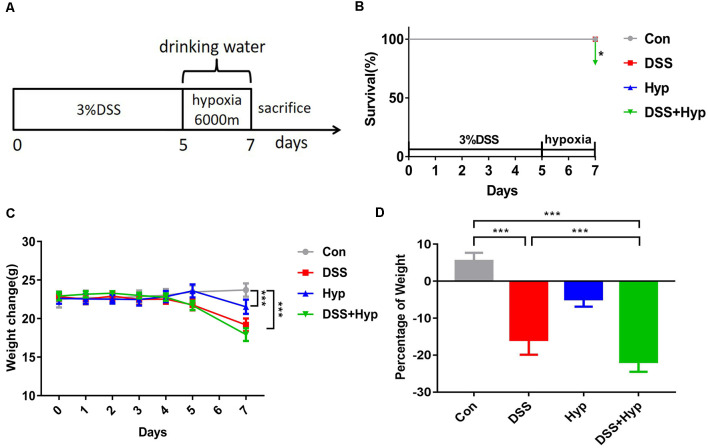
Hypoxia aggravated DSS-induced weight loss in mice. **(A)** Eight-week-old C57BL/6 mice were given 3% DSS in their drinking water for 5 days and/or hypoxia (6,000 m) for 2 days. The control mice were given water alone. On day 7, the mice were sacrificed and analyzed for colitis. **(B)** Survival rates of mice subjected to DSS, hypoxia, and DSS combined with hypoxia (*p < 0.05, *n* = 10–12/group). **(C)** Weights were monitored daily and normalized relative to the control animals (***p < 0.001, *n* = 10–12/group). **(D)** On day 7, the animals were sacrificed, and the weights were measured to assess the damage caused by hypoxia combined with DSS (***p < 0.001, *n* = 10–12/group).

**Figure 2:**
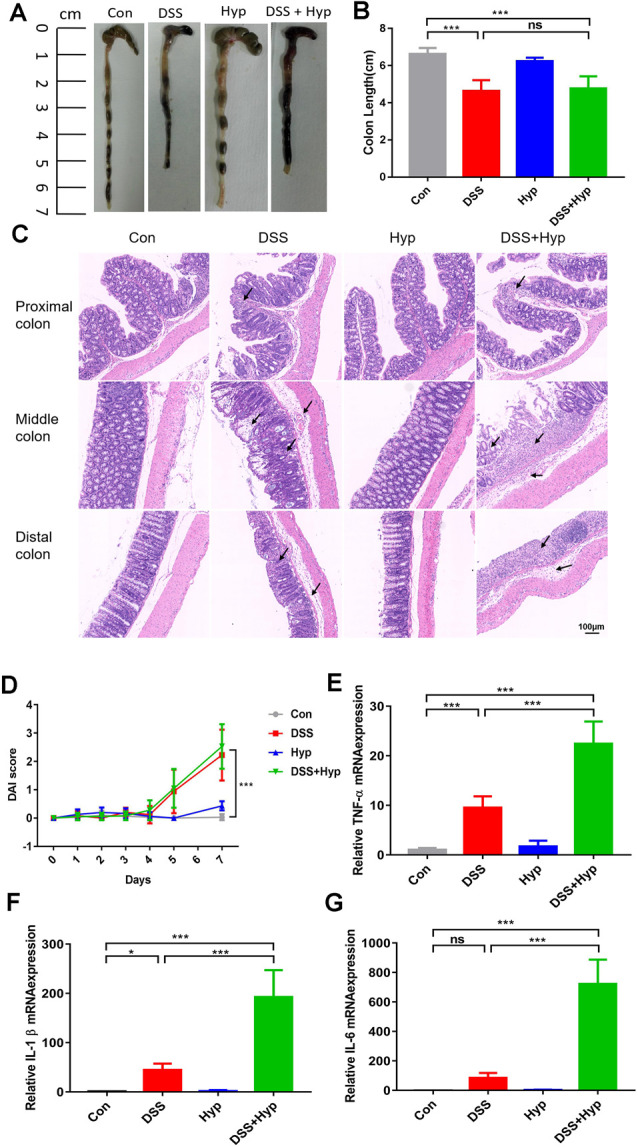
Hypoxia augmented the onset of colitis induced by DSS. On day 7, the animals were sacrificed, and the colons were excised and measured for assessment. **(A,B)** The outward appearance of the colon sections and colon length were analyzed (***p < 0.001, ns, not significant; *n* = 6/group). **(C)** H&E staining of the proximal, middle, and distal colon tissues isolated from the animals. **(D)** The disease activity index (DAI) was assessed (***p < 0.001, *n* = 10–12/group). **(E–G)** The inflammatory cytokines TNF-α, IL-1β and IL-6 were measured by real-time PCR (*p < 0.05, ***p < 0.001, ns, not significant; *n* = 6/group).

In our study, a well-characterized chemical model for initiating experimental IBD in mice was employed. In this model, mice were given 3% DSS in their drinking water *ad libitum* for five days. To study the effect of hypoxia on IBD, the mice were exposed to hypoxia (6,000 m, 2 days) after DSS treatment. The mice were sacrificed and examined for colitis on day 7 (Figure 1A). Our data show that the mice in both the DSS alone and DSS+ hypoxia groups developed severe diarrhea and colitis, which were characterized by loss of weight (Figure 1C), extensive ulceration of the epithelial layer, edema, and crypt damage of the bowel wall. Moreover, the mice in the DSS+ hypoxia group displayed more significant weight loss (Figure 1D), colon shortening (Figures 2A,B), and pathological injury, such as deterioration of the mucosal architecture with an almost complete loss of crypts (Figure 2C). The histological scores were significantly higher in the DSS+ hypoxia group than in the DSS alone group (Figure 2D). Also, increased expression of the inflammatory cytokines TNF-α, IL-1β, and IL-6 was observed by real-time PCR in the DSS+ hypoxia group compared with the DSS group (Figures 2E–G). The survival rate of the mice in the DSS+ hypoxia group decreased from 100% to 80% (Figure 1B). Such findings reveal that hypoxia augmented the onset of colitis induced by DSS.

### Hypoxia Increased the Serum Levels of IL-1β and IL-6 in DSS-Induced Colitis

**Figure 3:**
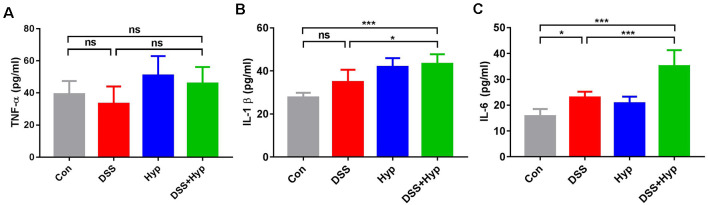
Hypoxia increased the serum levels of IL-1β and IL-6 in DSS-induced colitis. **(A–C)** Effects of DSS, hypoxia, and DSS combined with hypoxia on the serum levels of TNF-α, IL-1β, and IL-6. Hypoxia augmented the levels of IL-1β and IL-6 on day 7 under DSS treatment (*p < 0.05, ***p < 0.001, ns, not significant; *n* = 6/group).

Previous studies have suggested that colonic mucosal barrier function is damaged and colonic mucosal permeability is increased in mice with colitis (Gitter et al., 2001). It is important to study whether gut inflammatory cytokines enter the blood during the systemic inflammation induced by colitis. We analyzed the activation of systemic inflammation in the colitis mouse model. On day 7, after DSS + hypoxia treatment, the levels of IL-1β and IL-6 in the serum were markedly elevated (Figures 3B,C), while the levels of TNF-α did not change (Figure 3A). Moreover, there were significant differences in the pro-inflammatory cytokines IL-1β and IL-6 between the DSS + hypoxia group and the DSS group (Figures 3B,C). The data suggest that hypoxia amplifies the systemic inflammatory response in an IBD mouse model.

### Hypoxia Augmented the Activation of Microglia and Increased the Expression of Proinflammatory Cytokines in the Brains of Mice With Colitis

**Figure 4:**
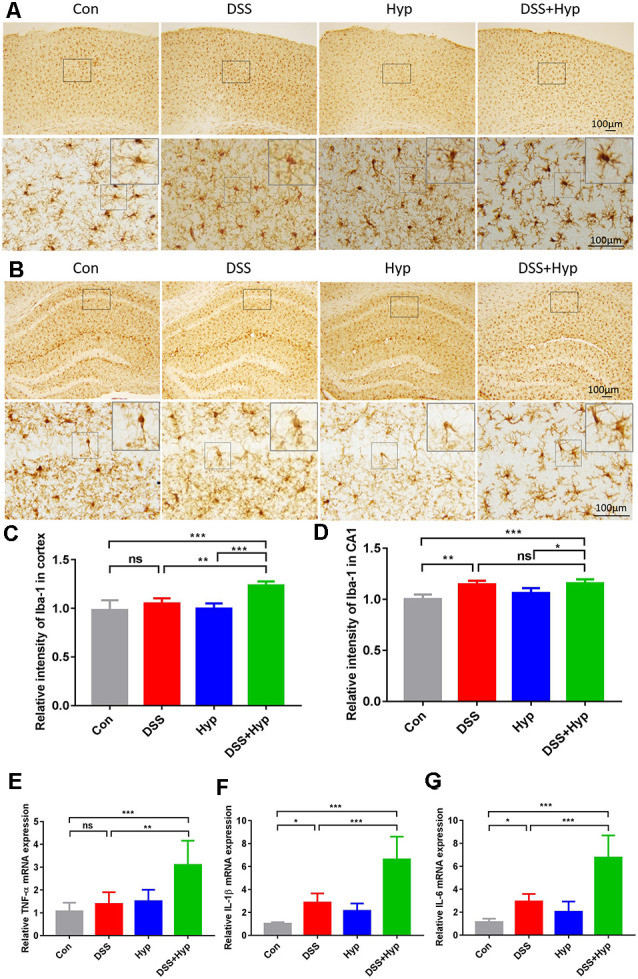
Hypoxia augmented the activation of microglia and increased the proinflammatory cytokines in the brains of mice with colitis. **(A,C)** Representative images and statistical analysis of microglial activation in the cortex on day 7 (**p < 0.01, ***p < 0.001, ns, not significant; *n* = 4/group). **(B,D)** Representative images and statistical analysis of microglial activation in the hippocampus on day 7 (*p < 0.05, **p < 0.01, ***p < 0.001, ns, not significant; *n* = 4/group). **(E–G)** Hypoxia + DSS and DSS increased TNF-α, IL-1β and IL-6 mRNA expression in the brain cortex (by real-time PCR). The expression of TNF-α, IL-1β and IL-6 was increased by hypoxia + DSS compared with DSS (*p < 0.05, **p < 0.01, ***p < 0.001, ns, not significant; *n* = 6/group).

The systemic pro-inflammatory response facilitates the development of cerebral injury during short-term hypoxia. To investigate the potential role of systemic inflammation in the development of brain inflammation during hypoxia, four groups of mice were exposed to normoxia or hypoxia (altitude of 6000 m): the control group, DSS group (3%), hypoxia group, and DSS+ hypoxia group. First, to evaluate brain inflammation in this model, we performed immunohistochemical staining for Iba-1 and analyzed the microglial population in the cortex and hippocampus. The microglial population in the cortex and hippocampus significantly increased after DSS + hypoxia-induced colitis (Figures 4A–D). Also, the number of microglia in the cortex tissues of the DSS + hypoxia group was higher than that of the DSS group (Figure 4C). The morphological changes and number of astrocytes were observed by GFAP immunohistochemical staining in the hippocampus. There was no difference in the morphology and number between DSS + hypoxia and DSS group (Supplementary Figure 1). Next, we measured the expression of proinflammatory cytokines in the brains on day 7. The levels of TNF-α, IL-1β, and IL-6 in the DSS + hypoxia group were increased on day 7 compared with those in the control group. Also, the levels of TNF-α, IL-1β and IL-6 mRNA in the brain were higher in the DSS + hypoxia group than in the DSS group (Figures 4E–G). These results demonstrate that hypoxia exposure for two days markedly increased the TNF-α, IL-1β, and IL-6 levels in both the serum and brain cortex in the colitis mouse model.

### Hypoxia Combined With DSS Downregulated Tight Junction Proteins

**Figure 5:**
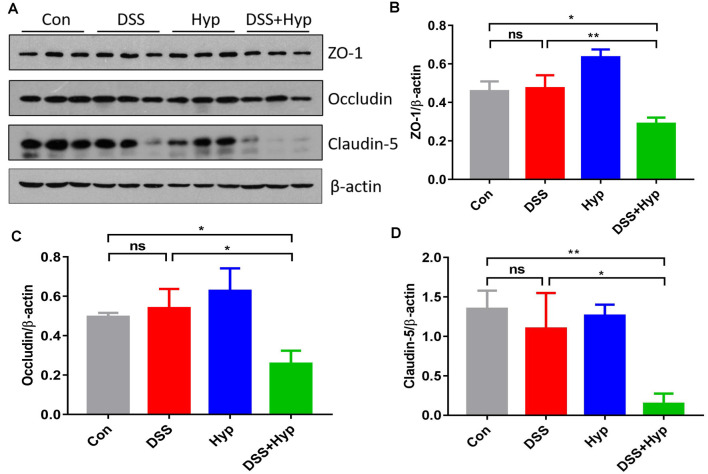
Hypoxia combined with DSS downregulated tight junction proteins. **(A–D)** Western blots and analysis of ZO-1, occludin, and claudin-5 expression in the brain cortex. Hypoxia + DSS decreased ZO-1, occludin and claudin-5 expression compared with control and DSS on day 7 (*p < 0.05, **p < 0.01, ns, not significant; *n* = 3/group).

The function of the BBB is maintained by TJs, and we assessed the TJs using western blotting. We evaluated the molecular changes in the TJs in the mouse brains. Although ZO-1, occludin, and claudin-5 did not exhibit marked changes after DSS treatment alone, ZO-1, occludin, and claudin-5 were downregulated in the mice that were subjected to DSS combined with hypoxia exposure. DSS + hypoxia treatment markedly decreased the ZO-1, occludin, and claudin-5 levels in the brain cortex compared with DSS treatment alone (Figures 5A–D). These results suggest that hypoxia accelerated brain injury by disrupting tight junctions in mice with DSS-induced colitis.

## Discussion

Hypoxia has long been associated with gastrointestinal dysfunction; hypoxia has also been proposed to be a risk factor for the development of neuroinflammation. However, whether hypoxia regulates neuroinflammation in colitis remains elusive. Here, we demonstrate that DSS + hypoxia resulted in abnormal expression of proinflammatory cytokines in the colon and plasma. Also, we showed increased expression of Iba-1, which is a marker of activated microglia, accompanied by increased expression of TNF-α, IL-1β, and IL-6 in the brain. Moreover, tight junction proteins were markedly downregulated.

In this study, we employed a well-characterized chemical model to initiate experimental IBD in mice by giving mice 3% DSS containing drinking water *ad libitum* for five days combined with exposure to hypoxic conditions for two days. In the present study, hypoxia may play a pro-inflammatory role initially in DSS-induced colitis. Hypoxia affected innate immune cells in the lamina propria of the intestine, neutrophils exhibited a delayed apoptosis rate, resulting in pathological injury induced by inflammation (Mecklenburgh et al., 2002). Furthermore, Hypoxic exposure increased the expression of pro-inflammatory cytokines and chemokines of DCs and mononuclear phagocytes (Blengio et al., 2013; Genua et al., 2014). Hypoxia can also elicit an inflammatory response in intestinal epithelial cells *via* the release of pro-inflammatory cytokines, which increased epithelial permeability during inflammatory responses (Taylor et al., 1998). Our study also showed that DSS combined with hypoxia treatment developed severe diarrhea and colitis characterized by loss of weight, colon shortening, and increase of inflammatory cytokines (Figure 2). Interestingly, under low-grade hypoxia exposure, intestinal epithelial cells exhibited an altered gene expression pattern, hypoxia induced the expression of epithelial-specific protein intestinal trefoil factor (ITF), thereby protecting against colitis in mouse models during hypoxia (Furuta et al., 2001). Also, HIF-1alpha-dependent induction of netrin-1 attenuated hypoxia-elicited inflammation at mucosal surfaces in mice (Rosenberger et al., 2009). In summary, hypoxia has a variety of functions under different environmental conditions including aggravating the injury and alleviating injury.

In the present study, we show that hypoxia exacerbated DSS-induced colitis and resulted in abnormal microglial activation and increased proinflammatory cytokine expression. Previously, we demonstrated that hypoxia augments LPS-induced brain inflammation and induces the development of cerebral edema in mice at high altitudes (Zhou et al., 2017). The mechanism is related to BBB disruption, activation of NF-κB and MAPKs, and reduced Na+-K+-ATPase activity (Song et al., 2016). Cytokines have been directly implicated in recent immunological studies, and they seem to have a crucial role in controlling inflammation (Neurath, 2014). Cytokines in the systemic circulation can also access the brain (Quan et al., 1999). In our study, the serum levels of IL-1β and IL-6 were increased (Figures 3B,C), and the levels of the proinflammatory cytokines TNF-α, IL-1β, and IL-6 in the brain were increased (Figures 4E–G). Researchers found that HIF-1α binds to HRE-like binding sites and upregulates IL-β, IL-6, and TNF-α during hypoxia exposure (Zhang et al., 2006). Therefore, in colitis induced by DSS combined with hypoxia, the upregulation of proinflammatory cytokines in the serum may be a key factor that results in neuroinflammation.

The present study evidenced that hypoxia treatment exacerbated DSS-induced colitis in the acute phase of inflammation. Here, we didn’t observe the effects on the chronic phase of inflammation. According to the literature, during chronic intestinal inflammation, serum IL-6 levels were up-regulated (Zonis et al., 2015). Recently, new research has shown that IBA-1 expressions were significantly increased in the cortex and hippocampus of the DSS-treated mice for 36 days (Zhang et al., 2020), reflects the neuroinflammation status. Additionally, pro-inflammatory cytokines, as well as levels of GFAP in the brain were up-regulated after DSS treatment. Meanwhile, Ki67 and DCX staining was significantly diminished in the hippocampus, indicating a decreased in hippocampal neurogenesis (Zonis et al., 2015). Effects of hypoxia on the chronic condition and the results in neuroinflammation are largely unknown.

One important mechanism by which peripheral inflammation affects CNS functions is through disruption of the BBB. In our experiment, the downregulation of the tight junction proteins ZO-1, occludin, and claudin-5 in the mouse brain was prominent in the group treated with DSS + hypoxia. TJs prevent protein diffusion and control cell trafficking from the blood to the CNS (Zlokovic, 2008), and the modification of TJ components may result in disease pathogenesis (Gonçalves et al., 2013). *In vitro* cell culture studies indicated that when oxygen-glucose deprivation (OGD) was combined with recombinant IL-1β exposure, the translocation of tight junction proteins and decreased transendothelial electrical resistance (TEER) were observed (Kangwantas et al., 2016). *In vivo*, rats pretreated with LPS alone for 11 h followed by exposure to hypobaric hypoxia for 1 h showed markedly increased Evans blue extravasation compared with rats subjected to LPS injection alone (Song et al., 2016), suggesting that LPS + hypoxia induces BBB disruption. Previous mouse experiments by our group also found that LPS-induced systemic inflammation accelerated the disruption of the BBB under hypoxia. We assessed tight junctions using transmission electron microscopy and western blotting, and the opening of tight junctions and the downregulation of occludin and VE-cadherin were observed in the mice that received LPS injection combined with hypoxia exposure (Zhou et al., 2017).

Gut microbiota is closely related to the immune system. The DSS model has been frequently used to mimic human colitis. Previous studies have shown that DSS-induced colitis in mice shows similarity in appearance to UC, and the quantification of the total amount of gut microbiota on colonic samples is significantly more after DSS treatment (Håkansson et al., 2015). The composition of the microbiota during colonic inflammation has also changed (Al-Bayati et al., 2018), a high proportion of pro-inflammatory species such as Enterobacteriaceae, Bacteroides fragilis, and Pseudomonas aeruginosa was found in patients with UC (Wang et al., 2007). Studies have found that intestinal flora disturbance is closely related to immune-mediated neurological diseases such as multiple sclerosis, Alzheimer’s disease, and Parkinson’s disease (Sampson et al., 2016; Cattaneo et al., 2017; Cekanaviciute et al., 2017). Microbiota can also influence outcomes after traumatic brain injury by altering in microbiota composition (Sundman et al., 2017). Therefore, in the present DSS-induced colitis model, whether hypoxia increases neuroinflammation by regulating the intestinal microecology needs further study.

In conclusion, the presence of hypoxia may enhance vulnerability to IBD, DSS plus hypoxia accumulated dramatically and promote the inflammatory reaction and the injury of TJs in the brain, compared with DSS or hypoxia alone. Our data provide new insight into the mechanisms underlying the association of hypoxia with excessive inflammatory responses and pathophysiological consequences in IBD-induced neuroinflammation. Thus, in this study, we proposed a relationship between IBD and brain inflammation under hypoxia. Understanding the hypoxia-associated signaling pathway may provide a new target for the prevention of severe high altitude-induced brain injury in IBD patients.

## Data Availability Statement

The raw data supporting the conclusions of this article will be made available by the authors, without undue reservation.

## Ethics Statement

The animal study was reviewed and approved by Animal Care and Use Committee of Institute of Basic Medical Sciences (No. IACUC-2017049).

## Author Contributions

LD, YH, HL and LZ conceptualized the study. LD, XL and YG prepared and maintained the mice. YH and LD designed and performed morphological analysis and biochemical assays. MZ, TZ, XC and MF provided the materials. YH wrote the manuscript. LZ, LG and LD discussed and edited the manuscript. LZ and HL supervised the project. All authors contributed to the article and approved the submitted version.

## Conflict of Interest

The authors declare that the research was conducted in the absence of any commercial or financial relationships that could be construed as a potential conflict of interest.

## Supplementary Material

SUPPLEMENTARY FIGURE 1 Astrocytes were observed by GFAP immunofluorescence staining in the hippocampus. **(A,B)** Representative images and statistical analysis of astrocytes were observed by GFAP immunofluorescence staining in the hippocampus CA1 on day 7 (*n* = 4/group).
